# Exploring the Associations between Perceived Organizational Support and Job Burnout among Chinese Academic Journal Editors: A Moderated Mediation Model

**DOI:** 10.3390/ijerph182212167

**Published:** 2021-11-19

**Authors:** Xiaoyan Yu, Shiyong Wu, Wei Chen, Wen Zheng, Mingxi Huang, Lei Yang, Shuyi Zhou

**Affiliations:** 1 Editorial Office of Modern Education Journal, South China Normal University, Guangzhou 510631, China; xiaoyan.yu@m.scnu.edu.cn; 2 South China Vocational Education Research Centre, South China Normal University, Foshan 528225, China; 3 School of Education, Huizhou University, Huizhou 516000, China; zhengwen@hzu.edu.cn; 4 School of Education, South China Normal University, Guangzhou 510631, China; huangmingxi@m.scnu.edu.cn; 5 School of Vocational Educational Teacher, Guangdong Polytechnic Normal University, Guangzhou 510665, China; echoyang@m.scnu.edu.cn; 6 Faculty of Foreign Language, Dongguan Science & Technology School, Dongguan 523470, China; 2019023366@m.scnu.edu.cn

**Keywords:** perceived organizational support, job burnout, job satisfaction, self-efficacy, Chinese academic journal editors, COVID-19

## Abstract

Background: Job burnout (JB) has become a prevalent emotional and psychological syndrome across diverse contexts, especially in the face of the COVID-19 pandemic. This study aimed to examine the relationship between perceived organizational support (POS), job satisfaction (JS), self-efficacy (SE), and JB, alongside their mechanism of interplay. Methods: We took 210 Chinese academic journal editors as the research participants and designed a moderated mediation model to examine the posited construct. All the data were gathered online and analyzed with the statistical software SPSS and SmartPLS. Results: The participants comprised 117 women (55.71%) and 93 men (44.29%). There were significant differences among observed variables in age, experience, and title. POS had a significant negative predictive effect on JB (95% CI = −0.43; −0.06). JS mediated the relationship between POS and JB (95% CI = −0.48; −0.11). SE moderated the association between JS and JB (95% CI = 0.04; 0.75) but did not function as a moderator in the relationship between POS and JS (95% CI = −0.01; 0.24). Conclusions: POS, JS, and SE were crucial determinants of JB among Chinese academic journal editors. Targeted interventions should be initiated to diminish editors’ feelings of being unappreciated, inefficient, dissatisfied, and unaccomplished at work.

## 1. Introduction

Burnout, widely characterized by three related, but empirically distinct, components—exhaustion, depersonalization, and reduced professional efficacy [1,2]—is an emotional and psychological syndrome prevalent throughout a variety of work contexts and job roles, such as the sport, education, parenting, and public service sectors [1,3,4,5,6]. Job burnout (JB) is influenced by a complex interplay between organizational and individual characteristics [6]. Concerning the organizational factor, an employee’s perceived organizational support (POS) has been significantly linked to decreasing levels of burnout [7]. Regarding the individual factors, personal attitude toward the whole job situation (e.g., job satisfaction, JS) and belief (e.g., self-efficacy, SE) have also been negatively associated with burnout [1,8].

POS refers to employees’ perceptions of the extent that their contributions are appreciated and their wellbeing is cared for by their organization [9]. Several burnout studies have shown that POS functions as a predictor of JB among health workers in Turkey [10], Chinese policemen [11], and Saudi Arabian healthcare staff [12]. JS is widely defined by Locke [13] as “a pleasurable or positive emotional state resulting from the appraisal of one’s job and job experience” and involves three dimensions: intrinsic, extrinsic, and general reinforcement [14]. Previous studies have also shown that JS is negatively related to JB [15,16,17,18]. SE refers to people’s judgments regarding their ability to succeed in a particular situation [19]. Employees with high SE are more effective at coping with negative emotions caused by work stress, such as JB.

Additionally, some theoretical and empirical studies have identified the interrelationship between those predictors of JB. According to social exchange theory (e.g., [20,21]), POS is a critical contributor to JS. Some researchers have noted that employees’ perceived and received organizational support positively predicts their levels of JS [22,23]. Moreover, according to the job demands–resources (JDR) model [24], when perceiving inefficient job resources (e.g., organizational support), employees tend to mobilize individual resources (e.g., SE) and are more likely to be satisfied with their work. Furthermore, some studies have demonstrated that SE controls the association between POS and JS [25,26], with a significantly negative relationship between SE, JS, and JB [3,27,28].

As part of a typical public service industry, academic journal editing is a stressful profession that involves numerous articles in print [29] alongside constant worries about accuracy in content and grammar [30,31], which can ultimately lead to job dissatisfaction [29] and JB [32]. This situation is similarly severe in the Chinese setting. Some Chinese scholars have identified that insufficient organizational support can lead to JB among this cohort [33]. In addition, Yu and Zhang [34] noted that emotional exhaustion had a significant effect on scientific journal editors’ physical and mental health and that JB was negatively related to their JS. Yao et al. [35] also found that editors’ mental health was positively associated with the level of support they received and was negatively associated with JB.

These previous studies have greatly enriched our understanding of people’s experiences with JB and its antecedents. However, these representative determinants affecting JB have not yet been integrated into a synthetic model that can identify individual and organizational factors. Specifically, little attention has been paid to the interaction between POS, JS, SE, and JB. More importantly, with the continuous spread of the COVID-19 pandemic and its restrictions, psychological problems, including burnout, have been further exacerbated [36,37,38,39]. Compared to those working in other disciplines, academic journal editors may suffer higher risks of burnout because of the precariousness of their position, defined by the ease of losing high-quality, article-format original scholarship and/or readership due to editing missteps [40]. Hence, there is great significance in investigating the influence of POS, JB, and SE on JB among academic journal editors facing the COVID-19 scenario.

The purpose of this study was to measure the predictive effects of POS, JS, and SE on JB, and explore the mechanisms at play among Chinese academic journal editors in the context of the COVID-19 outbreak. This study will add value to the theoretical literature on burnout constructs by assessing the complex relationship between JB and its correlations in a sample composed of Chinese academic journal editors. The following hypotheses were assumed:

**Hypotheses** **1** **(H1).** *POS negatively predicts JB.*

**Hypotheses** **2** **(H2).** *JS mediates the association between POS and JB.*

**Hypotheses** **3** **(H3).** *SE moderates the mediating impact of JS on the link between POS and JB.*

**Figure 1:**
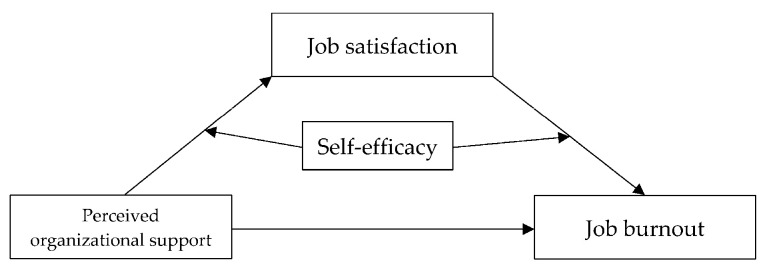
Posited model.

The complete hypothesized model is presented in Figure 1.

## 2. Materials and Methods

### 2.1. Participants

All participants were recruited online via each author’s social media (e.g., WeChat and email) due to the social-distancing restrictions under the COVID-19 pandemic. We first incorporated these four measures into a single file and then input the measures into the Wenjuanxing Online Survey Software, known as Chinese Qualtrics, so that participants could fill out the survey at any time and in any place. After the participants had completed the questionnaire, we examined the validity of the data and confirmed that all data collected between March and June 2021 were of high quality. Ultimately, 210 Chinese academic journal editors were included in our study.

### 2.2. Measures

#### 2.2.1. Perceived Organizational Support Scale

POS was calculated by the Chinese version of the eight-item scale extracted by Settoon et al. [41] from the short version of the Survey of Perceived Organizational Support used to evaluate the employee perception of the organization and actions that could affect their wellbeing [9]. Responses were rated on a 5-point Likert-type scale from 1 = strongly disagree to 5 = strongly agree. A sample item was “My organization cares about my opinions.” Higher scores reflect a higher level of POS. The Cronbach’s alpha for the English version was 0.94 [41,42], compared to a value of 0.83 for the Chinese version [43]. In the present study, the alpha coefficient is 0.89.

#### 2.2.2. Job Burnout Scale

JB was gauged with the Chinese version of the Maslach Burnout Inventory-Human Services Survey (MBI-HSS) originally developed by Maslach et al. [2] and comprised 22 items. As the most commonly adopted instrument for assessing occupational burnout, MBI-HSS consists of three subscales: emotional exhaustion (EE), depersonalization (DP), and personal accomplishment (PA). Responses are rated on a 7-point Likert-type scale (0 = never to 6 = everyday). Participants were asked to rate their agreements with statements such as “I feel burned out from my work.” Higher scores on the EE, DP, and diminished PA indicated a higher level of burnout. The Chinese version of the MBI-HSS has shown good reliability and validity in a Chinese context (e.g., [44,45]). The present study’s inter-item consistencies for EE, DP, PA, and the total items were 0.92, 0.85, 0.75, and 0.87, respectively.

#### 2.2.3. Job Satisfaction Scale

JS was assessed with the Chinese version of the Minnesota Satisfaction Questionnaire Short Form (MSQ-SF), originally constructed by Weiss, Dawis, and England [14], consisting of 20 items that examine “how people feel about different aspects of their jobs.” [46,47]. The MSQ-SF has three dimensions: intrinsic (12 items), extrinsic (8 items), and general (total items). Responses were rated on a 5-point Likert-type scale (1 = not satisfied, 5 = extremely satisfied). Participants were asked to rate their level of satisfaction with statements such as “The opportunities to work alone.” In this model, higher total scores indicate higher satisfaction with work. The Cronbach’s alphas of the original version’s ranges are 0.84–0.91, 0.77–0.82, and 0.87–0.92 for intrinsic, extrinsic, and general, respectively [14,48]. By contrast, the Cronbach’s alphas of the Chinese version’s scale ranges are 0.90–0.92, 0.83–0.84, and 0.91–0.93 for intrinsic, extrinsic, and general, respectively [49,50]. In the present study, the Cronbach’s alphas for the total, intrinsic, and extrinsic job satisfaction were determined as 0.94, 0.90, and 0.88, respectively.

#### 2.2.4. Self-Efficacy Scale

SE was measured with the Chinese version of the General Self-efficacy Scale (GSES), comprising ten items that investigate people’s self-beliefs in difficulties or dilemmas [51,52,53]. Responses were rated on a 4-point Likert-type scale (1 = absolutely incorrect, 4 = absolutely correct). Participants were asked to rate their degree of agreement with statements such as “I always succeed in solving the problem once I try my best.” In this model, higher scores indicate a higher sense of efficacy. The Cronbach’s alphas of the original version range from 0.75 to 0.91 [54,55], compared to 0.92 for the Chinese version [51,56]. In this study, the Cronbach’s alpha reliability coefficient was found to be 0.93.

### 2.3. Data Analysis

We deployed SmartPLS 3.3.2, the most widely used partial least square (PLS) statistical analysis software, and the PROCESS macro in SPSS to analyze the data. As our study relied on self-reported measures, we first checked the common method variance (CMV) using Harman’s one-factor test [57]. The outcomes indicated that the single factor solution explained only 35.23% of the variance (less than the recommended 40% threshold). Therefore, common method bias did not occur in this study. Multicollinearity was also assessed using the variance inflation factor (VIF). The results indicated that none of the variables exceeded the cut-off value of 5 suggested by Hair et al. [58]. The issue of CMV was, therefore, absent. Then, we evaluated the psychometric properties of the posited model by testing the reliability, convergent validity, and discriminant validity of the measurements. To improve the model’s satisfactory level, we retained the items with factor loadings exceeding 0.7 for further analysis [59]. Finally, the moderated mediation hypothesis was evaluated using a bootstrapping procedure with 5000 subsamples in PROCESS Model 58 [60]. We also produced a simple slope to display the difference in the conditional indirect effects across different levels of the moderator. The diagram indicated that significant effects were supported by the absence of zero within the confidence intervals.

### 2.4. Ethics

This study was examined and authorized by the South China Normal University Academic Ethics Committee. The research ethics approval was attached to the explanatory statement, and a consent form was supplied to the participants.

## 3. Results

### 3.1. Characteristics of Participants

The participants comprised 117 women (55.71%) and 93 men (44.29%). The ages of 72.38% of participants ranged from 31 to 50 years old. Years of work experiences spanned 1–5 (20.5%), 6–10 (31.4%), 11–20 (31.9%), and above 20 (16.2%) years. The majority of the participants (74.76%) obtained post-graduate degrees (M.A. = 49.05%, Ph.D. = 25.71%). Participants with the titles of professor, associate professor, lecturer, and primary accounted for 12.9%, 36.2%, 39.0%, and 11.9% of the total, respectively. The work contracts of the participants were both long-term (70%) and fixed-term (30%). Over half the participants worked as full-time editors (54.8%), compared with the dual roles of teacher–editor (27.1%) and manager–editor (18.1%). The journals employing the participants were found to be affiliated with universities (58.1%), research institutes (20%), industries (11.4%), and academic associations (10.5%). More detailed demographical information about journal publication scopes, categories, and metrics is provided in Table 1.

### 3.2. Descriptive Statistics

Table 2 presents the minimum values, maximum values, means, and standard deviations of variables. Editors obtained slightly higher scores than the median in POS and SE, indicating that they perceived high support from organizations and feelings of self-efficiency in work tasks. Similarly, the average JS of editors was higher than the median, meaning that the participants were, overall, satisfied with their jobs, especially at an intrinsic level, including factors such as job stability, responsibility, and self-directiveness. In terms of JB, the average value of editors was close to the median, which indicated that the participants had experienced medium job-related burnout, particularly JB due to emotional exhaustion and diminishing self-accomplishment.

Table 3 shows the significant differences observed between age, experience, and title. Regarding age differences, participants over 50 years old had the highest scores for JS and SE. The youngest participants, aged between 20 and 30 years old, scored highest for POS, while the cohort between 31 and 40 years old obtained the highest scores for JB, with the only significant difference (*p* = 0.01). In terms of experience, participants with the least work experience gained the highest scores for POS and SE, and editors over 20 years old scored the highest for JS. Participants who had 6 to 10 years of work experiences obtained the highest scores for JB, but significant differences were observed only for the latter two variables (*p* = 0.05 and 0.01). Regarding title, participants with professor-level titles gained the highest scores for JS and SE, while those who were awarded a primary title scored the highest for POS and JB. However, the difference was only significant for SE (*p* = 0.04). There were no significant differences found for the other control variables.

### 3.3. Correlation Analysis

Table 4 presents the correlations between the investigated variables and the Cronbach’s alpha coefficients of the scales. All the dimensions were significantly correlated with each other. The correlations were positive and significant for POS, JS, and SE (*p* < 0.01). JB was negatively significantly related with the other variables (*p* < 0.01). The two dimensions of JS both positively significantly predicted JS. All three dimensions of JB also positively significantly predicted JB, but low personal accomplishment was unrelated to emotional exhaustion and depersonalization. This result confirmed the suggestion that the professional efficacy subscale is weakly linked to the other two subscales and can be used separately [1,2,61].

### 3.4. Measurement Model Test

The measurement model was evaluated using the following criteria: factor loadings, composite reliability, Cronbach’s alpha, and average variance extracted (AVE), as suggested by Roldán and Sánchez-Franco [62]. The threshold values for factor loading, composite reliability, Cronbach’s alpha, and AVE are recommended to be greater than 0.7 [63], 0.7, 0.7, and 0.5, respectively [64]. However, MacCallum et al. [65,66] argued that an item value over 0.60 in a factor model with a small sample size is acceptable. Together, covering all dimensions, except for the low personal accomplishment factor loading exceeding 0.6, factors with values over 0.7 were extracted as observed variables, as shown in Table 5. The internal consistency reliabilities of latent variables ranged from 0.88 to 0.94 for Cronbach’s alpha and from 0.91 to 0.95 for composite reliabilities. All AVE values were also above the recommended 0.5 cut-off thresholds. Therefore, the modified measurement model presented adequate convergent validity.

Discriminant validity was also considered an essential index for testing the adequacy of the measurement model. SmartPLS 3.3 provides the heterotrait–monotrait (HTMT) ratio of correlations to assess discriminant validity. In accordance with Henseler et al. [67], the HTMT value should be below 0.85. The results indicated that all HTMT ratios between the two constructs were less than the recommended value (see Table 6). Hence, each construct was considered valid and distinct from other constructs.

### 3.5. Structural Model Test

The structural model was assessed by the following parameters using SmartPLS 3.3: the goodness of fit (GoF) indices, path coefficient, and *t*-value. The GoF criteria included the standardized root–mean–square residual (SRMR, <0.1 suggested by Kline [68]), squared Euclidean distance (d_ULS) and geodesic distance (d_G) (>0.05 recommended by Dijkstra and Henseler [69]), normed fit index (NFI, >0.8 advised by Hooper et al. [70]), and root mean squared residual covariance (RMS_theta, <0.12 advocated by Henseler et al. [71]). The statistical outcomes indicated that the estimated structural model had an appropriate model fit index (see Table 7).

According to Chin [72], the path coefficient beta value should be greater than 0.2, and the value of the *t*-statistics should be greater than 1.96. For convenience, we incorporated these two indicators into the moderated mediation analysis procedure used to perform the hypothesis test.

### 3.6. Moderated Mediation Test

**Figure 2:**
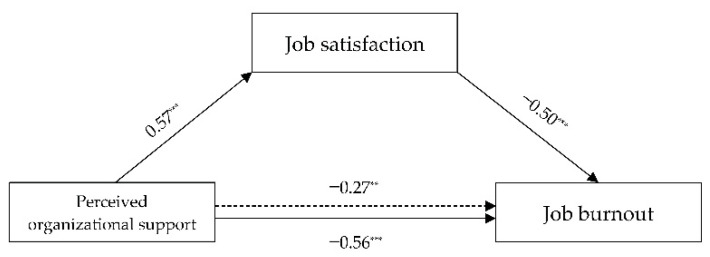
Mediation test. ** *p* < 0.01, *** *p* < 0.001.

We first used the PROCESS Model 4 to test the mediating role of JS. As shown in Table 8, POS significantly positively predicted JS (*β* = 0.57, *t* = 14.62, *p* < 0.001). Moreover, JS had a negative and significant effect on JB (*β* = 0.50, *t* = −4.14, *p* < 0.001). The indirect effect of POS on JB through JS was also significant (*β* = 0.29, *t* = −2.50, *p* < 0.01). The significant mediating effect was supported by the absence of zero within 95% confidence intervals [(−0.48, −0.11)]. The results indicated the existence of both a direct and an indirect effect (mediation). Therefore, JS partially mediated the relationship between POS and JB. The final mediating model explained 52% of the variance in JB (ab/c). Overall, hypotheses 1 and 2 were fully supported. Figure 2 shows the specific path coefficient among the variables.

**Figure 3:**
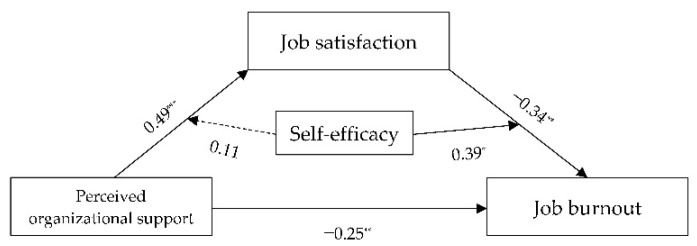
Moderated mediation test. * *p* < 0.05, ** *p* < 0.01, ***p < 0.001.

Next, we deployed the PROCESS Model 58 to test the total moderated mediating effects, following the suggestion of Hayes [73]. As shown in Table 9, in the direct path, POS negatively and significantly predicted JB (*β* = −0.25, *t* = −2.64, *p* < 0.01), indicating the presence of moderation. In the first half of the indirect path of moderation, POS positively and significantly predicted JS (*β* = 0.49, *t* = 12.81, *p* < 0.001); SE also positively significantly predicted JS (*β* = 0.32, *t* = 6.23, *p* < 0.00), but the interaction between POS and SE had no significant predictive effect on JS (*β* = 0.11, *t* = 1.83, *p* > 0.05). This result indicated that SE did not play a moderating role in the association between POS and JS. In the latter half of the indirect path of moderation, JS negatively and significantly predicted JB (*β* = −0.34, *t* = −2.63, *p* < 0.01). SE also negatively and significantly predicted JB (*β* = −0.46, *t* = −4.61, *p* < 0.001), and the interaction between JS and SE positively and significantly predicted JB (*β* = 0.39, *t* = 2.22, *p* < 0.05). These results suggest that SE functioned as a moderator in the relationship between JS and JB. Hypothesis 3 was, therefore, partially supported. Figure 3 depicts the specific path coefficients among the variables.

Furthermore, we performed a simple slope to visualize the moderating effect of SE on the correlation between JS and JB. With the increase in SE, the predictive effect of JS on JB gradually declined (from [effect = −0.56, *t* = −3.16, *p* < 0.01, 95% CI = −0.91; −0.21] to [effect = −0.12, *t* = −0.82, *p* > 0.05, 95% CI = −0.41; 0.17]).

**Figure 4:**
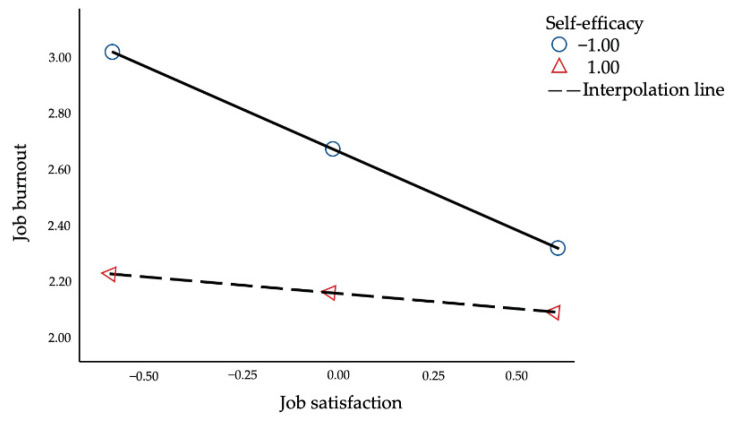
Simple plot image.

As shown in Figure 4, the conditional indirect effect of POS on JB through JS was statistically significant at the value of one standard deviation lower than the mean of self-efficacy (effect = −0.24, *SE* = 0.09, 95% CI = −0.43; −0.08). However, this indirect effect was not significant at the value of one standard deviation above the mean of the moderator (effect = −0.07, *SE* = 0.13, 95% CI = −0.33; 0.16). With an increase in the editors’ sense of SE, the indirect effect of POS on JB via JS was gradually weakened.

In sum, the moderated mediation model was partly established. JS acted as a mediator in the link between POS and JB. Moreover, SE served as a moderator in the association between JS and JB but did not play a moderating role in the relationship between POS and JS.

## 4. Discussion

In this study, we examined the moderating role of SE in the mediating effect of JS on the relationship between POS and JB among Chinese academic journal editors in the face of COVID-19. Participants’ overall means were intermediate or above the midpoint of the scales, indicating that they were highly appreciated by their organizations, quite satisfied with their work conditions, and self-efficacious when achieving their tasks. However, the participants felt burned out due to repetitive work. These results are consistent with the finding that scientific journal editors had moderate degrees of satisfaction with their jobs and felt burnt out [34]. The results are also similar to the findings indicating that newspaper editors suffered moderate rates of emotional exhaustion and high rates of personal accomplishment [74,75]. However, these results appear incongruent with the argument that the COVID-19 pandemic and its restrictions can aggravate employees’ feelings of burnout and fatigue [76,77]. This may be because being an academic journal editor is, by nature, a remote job that allows employees to work at home and online, thereby offering more flexibility, freedom, and safety in the case of COVID-19. Consequently, the current pandemic did not produce extra levels of anxiety and stress regarding the mandatory lockdowns, loss of income, or fear of unemployment and thus failed to accelerate their levels of burnout.

Furthermore, the significant difference analysis revealed that 31–40-year-old employees with 6–10 years of work experience had higher levels of burnout than their peers, similar to the empirical findings of Reinardy [78]. Except for differences in age, experience, and title, no significant differences were found in the control variables such as gender, position, or institute, unlike the findings asserting that employees who are female [35,79], work at a small firm, and/or work as a copy editor [75,80,81,82,83,84,85] are more vulnerable to burnout than others. This result indicated that middle-aged academic journal editors who struggled in their early careers were more likely to experience burnout. Hence, more job resources and support should be provided for these editors to enhance their SE and JS and mitigate their JB.

In addition, the results presented close links between POS, JS, SE, and JB among Chinese academic journal editors. The specific interrelationship between the four observed variables is discussed explicitly in the following subsections.

### 4.1. The Relationship between POS and JB

The results indicated that POS had a significant negative correlation with all dimensions of JB and could significantly and negatively predict JB. This indicates that the higher the POS levels of Chinese academic journal editors, the lower their JB.

This result is consistent with previous theoretical arguments [9,86]. According to the JDR model [7], organizational support is essential in minimizing JB syndrome. Moreover, providing employees with necessary organizational support can help employees effectively tackle strained work situations [87]. When perceiving desirable levels of support from their organizations, employees may feel more effective and less monotonous when coping with repetitive work tasks. Conversely, reducing POS may increase employees’ JB. This study also found that, regardless of their characteristics, Chinese academic journal editors who perceived support from their organizations had a comparatively low level of JB. This result is also in line with the empirical findings that reported a negative correlation between POS and all JB components—namely, the higher the POS, the lower the JB [88,89,90,91]. For instance, Reinardy [82] found that broadcast journalists perceiving a low level of organizational support had significantly higher levels of burnout. Based on these findings, it can be inferred that POS is a vital predictor for JB that should be considered when committing to diminish levels of JB among Chinese academic journal editors. Thus, affected stakeholders, including journals, industries, associations, and universities, can mitigate editors’ JB by providing the organizational support that editors want. These stakeholders also need to consider the dynamics and diversity of organizational support by improving working environments, raising wages, and ameliorating the setbacks that would be detrimental to position promotion and work accomplishment.

### 4.2. The Mediating Role of JS

The results confirmed that JS could act as a mediator between POS and JB among Chinese academic journal editors; that is, the impact of POS on JB could be partly direct and partly indirect, through JS.

This result is congruent with the findings that the organizational support perceived by employees plays a critical role in predicting and promoting JS [92,93]. Employees with high levels of POS presented greater satisfaction with their jobs [86]. According to social exchange theory [20,21], when perceiving strong support from an organization, employees tend to reciprocate by engaging in their jobs and developing loyalty to their organization [25]. In comparison, employees feeling unsupported are likely to feel dissatisfied with their jobs and reduce their commitment to the organization [94]. Therefore, when the organization’s assistance meets Chinese academic journal editors’ needs for recognition, the JS will increase among this group of workers [29].

This result also agrees with the assumption of Maslach, Schaufeli, and Leiter [1] that a drop in satisfaction serves as a precursor to burnout. Likewise, Cook and Banks [95] found that JS was significantly negatively related to JB among copy editors working at a small newspaper in the United States. This result is also in line with Liu and Lo [96], who stated that JS is negatively related to JB among Taiwanese reporters and acts as a mediator in burnout and turnover intention.

Academic journal editors experience an increased risk of stress in their profession due to deadlines, the need for a low rate of word inaccuracy, and ongoing updates to technology [97]. This situation is much more challenging for Chinese academic journal editors, for whom the rate of acceptable content inaccuracy was decreased from below 0.03% to below 0.02% in the latest Regulation on the Quality of Newspapers and Journals [98]. Moreover, these editors feel stressed and exhausted about the annual journal evaluation outcomes from assessment institutes, which determine whether their journals will be abstracted by the Chinese Social Science Citation Index and the Social Science Citation Index of Web of Science. A higher ranking can bring many benefits to journals, such as greater financial support and faster promotions for editors. However, editors who work at ordinary journals may have fewer opportunities to realize their own career aspirations, with a high risk of being fired. Many editors have expressed strong intentions to leave the article-publishing industry, with high levels of emotional exhaustion and depersonalization, and low levels of personal accomplishment [99]. Therefore, industry practitioners should consider the importance of avoiding academic journal editor JB by improving editors’ satisfaction with their overall job situation and promoting the availability of organizational support. When academic journal editors have strong perceptions of their contributions being valued and their wellbeing being cared for, the editors are more satisfied with their work and can overcome the feelings of burnout.

However, it is worth noting that POS still had a significant direct effect on JB, which indicated that JS was only a partial mediator. Complete mediation can be established in theory but would be rare. Moreover, there may be other variables that were not considered in this study.

### 4.3. The Moderating Role of SE

This study also indicated that, in the indirect effect of POS on JB via JS, SE significantly moderated only the latter half of the relationship between JS and JB. The conditional effect outcome showed a transitional point along the continuum of the moderator between statistically significant and nonsignificant. Specifically, when Chinese academic journal editors’ sense of SE was low, the negatively predictive effect of JS on JB was strong; when the Chinese academic journal editors’ sense of SE was high, the negatively predictive effect of JS on JB was weakened.

This result further confirmed the social cognitive theory [56,100], which theorizes that both individual disposition and organizational factors influence work performance and attitude. This result also coincides with the argument that employees’ inefficacy may lead to job dissatisfaction and burnout [4,100]. In this study, Chinese academic journal editors’ SE significantly negatively moderated the effect of JS on JB. However, we noted that the moderating role of SE at a low level was more robust than that at a high level. This result could be due to the protective effect of SE [101,102]. When employees perceive that they have efficient organizational support and feel satisfied with their work, which can significantly overcome feelings of burnout, those employees are prone to mobilizing fewer individual resources such as SE. However, if Chinese academic journal editors are dissatisfied with their organizational support and job conditions, they are at high risk of experiencing JB even with a high level of SE. Therefore, the organizations should focus on individual-level intervention strategies to enhance employees’ work-related abilities to cope with work stressors [34], including the development of coping skills [103], the promotion of healthy work–life patterns [104], and the restructuring of irrational beliefs [105].

However, the present results do not completely support the JDR model, which proposes that POS influences JS through SE [24]. Unlike the previous finding that employees’ SE could significantly promote the positive effect of POS on JS [11], the results in the current research instead indicate that Chinese academic journal editors’ SE had no moderating effect on the predictive effects of POS on JS. Given the relatively low correlation between SE and POS (*r* = 0.32), this remarkable result could be explained by the social cognitive theory that proposes that, when employees lack confidence in their abilities to achieve their work aspirations or lack the desired opportunities and support needed to take responsibility and increase their dedication, those workers will feel unaccomplished and dissatisfied with their jobs. Therefore, despite the gap between the hypothesized model and the empirical path, organizations also need to focus on organizational-level intervention strategies, involving reducing workload [106], improving the organizational climate [107], and identifying and providing the personalized and diverse support that employees desire. These strategies will increase confidence among employees in successfully fulfilling their work obligations and further improve their JS.

## 5. Limitations and Further Work

While the reliability and validity issues of the model were verified in the current study, there are a few limitations that must be acknowledged. The first limitation is that the data were gathered by self-reporting. Therefore, causality and generalization in the results should be treated with caution. Nevertheless, the results suggest common scenarios among Chinese academic journal editor cohorts. The second limitation is that although the sample was deemed representative, the sample size was still small, constraining the generalizability of the findings. Further research is needed to enlarge the sample size and improve the statistical effect size post-COVID-19. The third limitation is that we did not consider controlled variables when validating the model, which may have resulted in statistical bias. Future work should incorporate more controlled variables, such as age, title, and work experience, which show differences in the levels of their constructs, to enhance the statistical power and model discrimination.

## 6. Conclusions

This study aimed to design a moderated mediation model in which POS, JS, SE, and JB were tested among Chinese academic journal editors, drawing on social cognitive theory, social exchange theory, and the JDR model. The results revealed that POS is a critical antecedent when predicting JB and JS. The results also showed that JS partially mediated the impact of POS on JB. Although SE only moderated the relationship between JS and JB, the total moderating effect was significant. Notably, the moderating role of SE at the low level was stronger than that at a high level. This empirical result adds value to the current literature on employees’ emotional and psychological health in the academic publishing industry. This research also provides an informative insight into how to provide academic journal editors with their desired level of organizational support, accelerate their SE, and boost their satisfaction with their jobs in the face of the COVID-19 pandemic by offering more opportunities to develop their professional skills and more flexible career promotion options. This study could be further strengthened and generalized by amplifying the sample size and diversity to increase the effect size and decrease random errors.

## Figures and Tables

**Table 1:** Characteristics of study participants.

Characteristics	Sub-Characteristics	Number	Percentage
Gender	Male	93	44.3%
	Female	107	55.7%
Age	20–30	20	9.5%
	31–40	80	41.9%
	41–50	64	30.5%
	Over 50	38	18.1%
Experience	1–5	43	20.5%
	6–10	66	31.4%
	11–20	67	31.9%
	Over 20	34	16.2%
Degree	Doctorate	54	25.7%
	Master’s	103	49.0%
	Bachelor’s	50	23.8%
	College	3	1.4%
Title	Professor	27	12.9%
	Associate professor	76	36.2%
	Lecturer	82	39.0%
	Primary	25	11.9%
Role	Editor	115	54.8%
	Teacher and Editor	57	27.1%
	Manager and Editor	38	18.1%
Affiliation	University	122	58.1%
	Institute	42	20.0%
	Industry	24	11.4%
	Association	22	10.5%
Contract	Long term	146	69.5%
	Fixed term	64	30.5%
Discipline	Social science	79	37.6%
	Nature science	81	38.6%
	Cross-discipline	50	23.8%
Category	Multiple	46	21.9%
	Specific	117	55.7%
	University journals	47	22.4%
Metrics	Chinese Top-tier	114	54.3%
	Web of Science Core Collection	8	3.8%
	Others	88	41.9%

**Table 2:** Minimum, maximum, average, and standard deviation of variables and sub-variables.

Variables	Minimum	Maximum	Average	Standard Deviation
Perceived organizational support	1.25	5	3.22	0.71
Job satisfaction	1.6	5	3.35	0.57
Intrinsic satisfaction	1.83	5	3.47	0.56
Extrinsic satisfaction	1.25	5	3.18	0.67
Self-efficacy	1.1	4	2.6	0.55
Job burnout	0.64	4.91	2.44	0.83
Emotional Exhaustion	0	6	2.52	1.3
Depersonalization	0	6	1.69	1.21
Low personal accomplishment	0.13	5.13	2.82	0.95

**Table 3:** Significant differences in perceived organizational support, job satisfaction, self-efficacy, and job burnout in age, experience, and title.

Variables	Age										Experience										Title									
	20–30 (*n* = 20)		31–40 (*n* = 88)		41–50 (*n* = 64)		50 Above (*n* = 38)		*F*	*Sig.*	1–5 (*n* = 43)		6–10 (*n* = 66)		11–20 (*n* = 67)		20 Above (*n* = 34)		*F*	*Sig.*	Professor (*n* = 27)		A/P (*n* = 76)		Senior (*n* = 82)		Primary (*n* = 25)		*F*	*Sig.*
	*M*	*SD*	*M*	*SD*	*M*	*SD*	*M*	*SD*			*M*	*SD*	*M*	*M*	*SD*	*M*	*SD*	*M*			*M*	*SD*	*M*	*SD*	*M*	*SD*	*M*	*SD*		
POS	3.29	0.63	3.21	0.78	3.21	0.68	3.22	0.68	0.07	0.98	3.34	0.65	3.05	0.7	3.3	0.74	3.22	0.71	1.93	0.13	3.22	0.78	3.21	0.73	3.17	0.69	3.40	0.67	0.64	0.59
JS	3.27	0.63	3.33	0.57	3.32	0.58	3.51	0.51	1.3	0.28	3.38	0.59	3.2	0.52	3.43	0.59	3.47	0.54	2.66	0.05 *	3.53	0.64	3.37	0.57	3.28	0.53	3.35	0.60	0.70	0.55
SE	2.6	0.59	2.55	0.53	2.58	0.59	2.75	0.52	1.21	0.31	2.67	0.62	2.5	0.55	2.62	0.52	2.66	0.53	1.11	0.35	2.87	0.58	2.60	0.56	2.54	0.52	2.51	0.54	2.74	0.04 *
JB	2.54	0.56	2.56	0.88	2.48	0.8	2.03	0.77	4.05	0.01 **	2.47	0.82	2.69	0.82	2.28	0.85	2.21	0.72	3.84	0.01 *	2.27	0.88	2.39	0.79	2.49	0.79	2.60	1.01	0.86	0.46

* *p* < 0.05, ** *p* < 0.01; POS: perceived organizational support, JS: job satisfaction, SE: self-efficacy, JB: job burnout, A/P: associate professor, *n*: number, *M*: mean, *SD*: standard deviation.

**Table 4:** Correlations of variables and sub-variables.

Variables	1	2	3	4	5	6	7	8	9
Perceived organizational support	(0.90)								
Job satisfaction	0.71 **	(0.94)							
Intrinsic satisfaction	0.62 **	0.95 **	(0.90)						
Extrinsic satisfaction	0.73 **	0.92 **	0.76 **	(0.88)					
Self-efficacy	0.32 **	0.48 **	0.53 **	0.35 **	(0.93)				
Job burnout	−0.48 **	−0.51 **	−0.50 **	−0.45 **	−0.45 **	(0.87)			
Emotional Exhaustion	−0.46 **	−0.43 **	−0.38 **	−0.43 **	−0.29 **	0.86 **	(0.92)		
Depersonalization	−0.33 **	−0.30 **	−0.29 **	−0.27 **	−0.21 **	0.79 **	0.67 **	(0.85)	
Low personal accomplishment	−0.19 **	−0.33 **	−0.39 **	−0.21 **	−0.48 **	0.44 **	−0.00	0.08	(0.75)

** *p* < 0.01; Cronbach’s alpha coefficients are presented in parentheses diagonally.

**Table 5:** Factor loading, Cronbach’s alpha, composite reliability, and average variance of the items extracted from the variables.

Constructs	Items	Factor Loading	Cronbach	Composite Reliability	Average Variance Extracted
Perceived organizational support	Perceived organizational support 1	0.82	0.88	0.91	0.63
	Perceived organizational support 2	0.83			
	Perceived organizational support 3	0.88			
	Perceived organizational support 5	0.74			
	Perceived organizational support 7	0.76			
	Perceived organizational support 8	0.74			
Job satisfaction	Intrinsic 4	0.73	0.91	0.92	0.54
	Intrinsic 7	0.71			
	Intrinsic 9	0.77			
	Intrinsic 10	0.79			
	Intrinsic 11	0.75			
	Intrinsic 12	0.81			
	Extrinsic 2	0.75			
	Extrinsic 3	0.76			
	Extrinsic 8	0.70			
Self-efficacy	Self-efficacy 1	0.75	0.94	0.95	0.69
	Self-efficacy 4	0.78			
	Self-efficacy 5	0.84			
	Self-efficacy 6	0.85			
	Self-efficacy 7	0.87			
	Self-efficacy 8	0.86			
	Self-efficacy 9	0.87			
	Self-efficacy 10	0.81			
Job burnout	Emotional exhaustion 3	0.87	0.93	0.95	0.78
	Emotional exhaustion 4	0.89			
	Emotional exhaustion 5	0.93			
	Emotional exhaustion 6	0.82			
	Emotional exhaustion 7	0.90			
	Depersonalization 2	0.70			
	Depersonalization 3	0.67			
	Lack of Personal Accomplishment 4	0.61			
	Lack of Personal Accomplishment 7	0.60			

**Table 6:** Heterotrait–monotrait (HTMT) discrimination validity of the measurement model.

Variables	1	2	3	4
Perceived organizational support	-			
Job satisfaction	0.79	-		
Self-efficacy	0.32	0.46	-	
Job burnout	0.55	0.56	0.49	-

**Table 7:** Fit indices of the structural model.

Fit index	SRMR	d_ULS	d_G	NFI	RMS_theta
Proposed value	<0.10	>0.05	>0.05	>0.80	<0.12
Estimated value	0.07	2.21	0.93	0.85	0.10

SRMR: standardized root mean square residual, d_ULS: squared Euclidean distance, d_G: geodesic distance, NFI: normed fit index, RMS_theta: root mean squared residual covariance.

**Table 8:** Results of the mediating effect of job satisfaction on the relationship between perceived organizational support and job burnout.

Path		Overall Model Fit			Regression Coefficient Significance			
Outcome	Predictor	*R*	*R* ^2^	*F*	*β*	*t*	LLCI	ULCI
JS	POS	0.71	0.51	213.84 ***	0.57	14.62 ***	0.49	0.65
JB	JS	0.54	0.29	41.93 ***	−0.50	−4.14 ***	−0.74	−0.26
	POS				−0.27	−2.79 **	−0.46	−0.08
Total effect		0.48	0.23	61.91 ***	−0.56	−7.87 ***	−0.70	−0.42
Indirect effect					−0.29	−2.50 **	−0.48	−0.11

** *p* < 0.01, *** *p* < 0.001; POS: perceived organizational support, JS: job satisfaction, JB: job burnout, LLCI: lower limit confidence interval, ULCI: upper limit confidence interval.

**Table 9:** Results of the moderating effect of self-efficacy on the mediating role of job satisfaction in the relationship between perceived organizational support and job burnout.

Regression Equation		Overall Model Fit			Regression Coefficient Significance			
Outcome	Predictor	*R*	*R* ^2^	*F*	*β*	*t*	LLCI	ULCI
JS	POS	0.77	0.59	96.89 ***	0.49	12.81 ***	0.41	0.56
	SE				0.32	6.23 ***	0.22	0.41
	POS*SE				0.11	1.83	−0.01	0.24
JB	JS	0.60	0.36	23.37 ***	−0.34	−2.63 **	−0.59	−0.09
	POS				−0.25	−2.64 **	−0.43	−0.06
	SE				−0.46	−4.61 ***	−0.66	−0.27
	JS*SE				0.39	2.22 *	0.04	0.75

* *p* < 0.05, ** *p* < 0.01, *** *p* < 0.001; POS: perceived organizational support, JS: job satisfaction, SE: self-efficacy, JB: job burnout, LLCI: lower limit confidence interval, ULCI: upper limit confidence interval.

## Data Availability

The data that support the findings of this study are available from the corresponding author with the permission of South China Normal University, upon request. Restrictions apply to the availability of these data, which were used under license for this study.
